# Efficacy and safety of topical and systemic medications: a systematic literature review informing the EULAR recommendations for the management of Sjögren’s syndrome

**DOI:** 10.1136/rmdopen-2019-001064

**Published:** 2019-10-28

**Authors:** Pilar Brito-Zerón, Soledad Retamozo, Belchin Kostov, Chiara Baldini, Hendrika Bootsma, Salvatore De Vita, Thomas Dörner, Jacques-Eric Gottenberg, Aike A. Kruize, Thomas Mandl, Wan-Fai Ng, Raphaele Seror, Athanasios G. Tzioufas, Claudio Vitali, Simon Bowman, Xavier Mariette, Manuel Ramos-Casals

**Affiliations:** 1 Autoimmune Diseases Unit, Department of Medicine, Hospital CIMA-Sanitas, Barcelona, Spain; 2 Laboratory of Autoimmune Diseases Josep Font, CELLEX, IDIBAPS, Barcelona, Spain; 3 Department of Rheumatology, Instituto Modelo de Cariología Privado S.R.L, Instituto Universitario de Ciencias Biomédicas de Córdoba, Cordoba, Argentina; 4 Instituto De Investigaciones En Ciencias De La Salud (INICSA), Universidad Nacional de Córdoba (UNC), Cordoba, Argentina; 5 Research Primary Healthcare Transversal Research Group, CAP Les Corts, CAPSBE, IDIBAPS, Barcelona, Spain; 6 Statistics and Operations Research Department, Universitat Politecnica de Catalunya, Barcelona, Spain; 7 Rheumatology Unit, Universita degli Studi di Pisa, Pisa, Italy; 8 Department of Rheumatology and Clinical Immunology, University Medical Center Groningen, University of Groningen, Groningen, The Netherlands; 9 Clinic of Rheumatology, University Hospital Santa Maria della Misericordia, Udine, Italy; 10 Department of Medicine/Rheumatology and Clinical Immunology and DRFZ, Charité Universitätsmedizin Berlin Campus Charite Mitte, Berlin, Germany; 11 Department of Rheumatology, Strasbourg University Hospital, National Reference Center for Rare Systemic Autoimmune Diseases, CNRS, IBMC, UPR 3572, Université de Strasbourg, Strasbourg, France; 12 Department of Rheumatology and Clinical Immunology, University Medical Center Utrecht, Utrecht, The Netherlands; 13 Department of Rheumatology, Skane University Hospital Malmö, Lund University, Lund, Sweden; 14 NIHR Newcastle Biomedical Research Centre, Newcastle upon Tyne NHS Foundation Trust, Newcastle upon Tyne, UK; 15 Department of Rheumatology, Assistance Publique – Hôpitaux de Paris, Hôpitaux Universitaires Paris-Sud, Le Kremlin-Bicêtre, Le Kremlin-Bicetre, France; 16 Center for Immunology of Viral Infections and Autoimmune Diseases, INSERM UMR 1184, Université Paris-Sud, Université Paris-Saclay, Paris, France; 17 Department of Pathophysiology, School of Medicine, National and Kapodistrian University of Athens, Athens, Greece; 18 Villa San Giuseppe, Istituto S. Stefano, Como, Italy; 19 Department of Rheumatology, University Hospitals Birmingham NHS Foundation Trust, Birmingham, UK; 20 Department of Autoimmune Diseases, ICMiD, Hospital Clinic de Barcelona, Barcelona, Spain

**Keywords:** Sjøgren's syndrome, treatment, multidisciplinary team-care

## Abstract

**Objective:**

To evaluate current evidence on the efficacy and safety of topical and systemic medications in patients with primary Sjögren syndrome (SjS) to inform European League Against Rheumatism treatment recommendations.

**Methods:**

The MEDLINE, EMBASE and Cochrane databases were searched for case-control/prospective cohort studies, randomised controlled trials (RCTs) and systematic reviews.

**Results:**

Current evidence in primary SjS patients fulfilling the 2002 criteria is based on the data from 9 RCTs, 18 prospective cohort studies and 5 case-control studies. Two Cochrane systematic literature reviews (SLRs) have reported that topical treatments for dry mouth and dry eye are safe and effective. Ocular cyclosporine A was safe and effective in two RCTs including 1039 patients with dry eye syndrome. Two Cochrane SLRs on serum tear drops and plugs showed inconsistency in possible benefits, both for symptoms and objective measures. Five RCTs reported significant improvements in oral dryness and salivary flow rates for pilocarpine and cevimeline. An RCT showed no significant placebo-differences for hydroxychloroquine 400 mg/day for the primary outcome (visual analogue scale (VAS) composite of dryness, fatigue and pain). We identified seven RCTs carried out in primary SjS patients. RCTs using infliximab, anakinra and baminercept found no placebo-differences for the primary outcomes. The two largest RCTs randomised 255 patients to receive rituximab or placebo and reported no significant results in the primary outcome (VAS composite), while prospective studies suggested efficacy in systemic disease.

**Conclusion:**

The current evidence supporting the use of the main topical therapeutic options of primary SjS is solid, while limited data from RCTs are available to guide systemic therapies.

**Key messages**

**What is already known about this subject?**

- EULAR has issued the 2019 recommendations for the management of Sjögren syndrome.

**What does this study add?**

- The current evidence supporting the efficacy and safety of the main topical therapeutic options for the treatment of the sicca symptoms of primary Sjögren’s syndrome (SjS) is solid.
- There is no information on the differential efficacy and safety of the main systemic therapeutic options available.
- Limited data are available from controlled trials to guide systemic treatment

**How might this impact on clinical practice?**

- This systematic literature review informed the task force for the ‘EULAR recommendations for the management of Sjögren syndrome’ that will help guide practice for physicians from several medical specialties involved in the management of the disease.

## Introduction

Sjögren’s syndrome (SjS), a chronic, systemic autoimmune disease, has no cure. Although it was identified as a disease more than a century ago,^1^ the therapeutic management has not changed significantly in recent decades.^2^ The specific pathogenic basis of a disease that targets the exocrine glands has led to a very specific type of therapy (agents locally applied to the mucosal surfaces involved) as one of the key approaches. In contrast, the systemic element of SjS has traditionally been tackled using glucocorticoids (GCs) and immunosuppressive agents, due to their use in similar systemic diseases such as systemic lupus erythematosus or vasculitis.

During the first decade of this century, Food and Drug Administration (FDA) approval of muscarinic agents and topical cyclosporine A (CyA) for oral and ocular dryness in SjS patients, respectively, and the first studies testing biological agents were considered the first signs of a new, game-changing therapeutic scenario for primary SjS patients. Despite this, the first specific systematic literature review (SLR) of SjS therapy, published in 2010, found that evidence remained very limited, without solid results that could change the disease management.^3^ Since 2010, there have been significant advances, including the accurate characterisation and scoring of the disease burden,^4 5^ the patient-centred therapeutic response^6^ and the publication of large, well-designed therapeutic studies.^7^

The aim of this review was to inform the new EULAR recommendations on the current state of evidence on the efficacy and safety of the main topical and systemic therapies used in SjS.

## Methods

**Figure 1:**
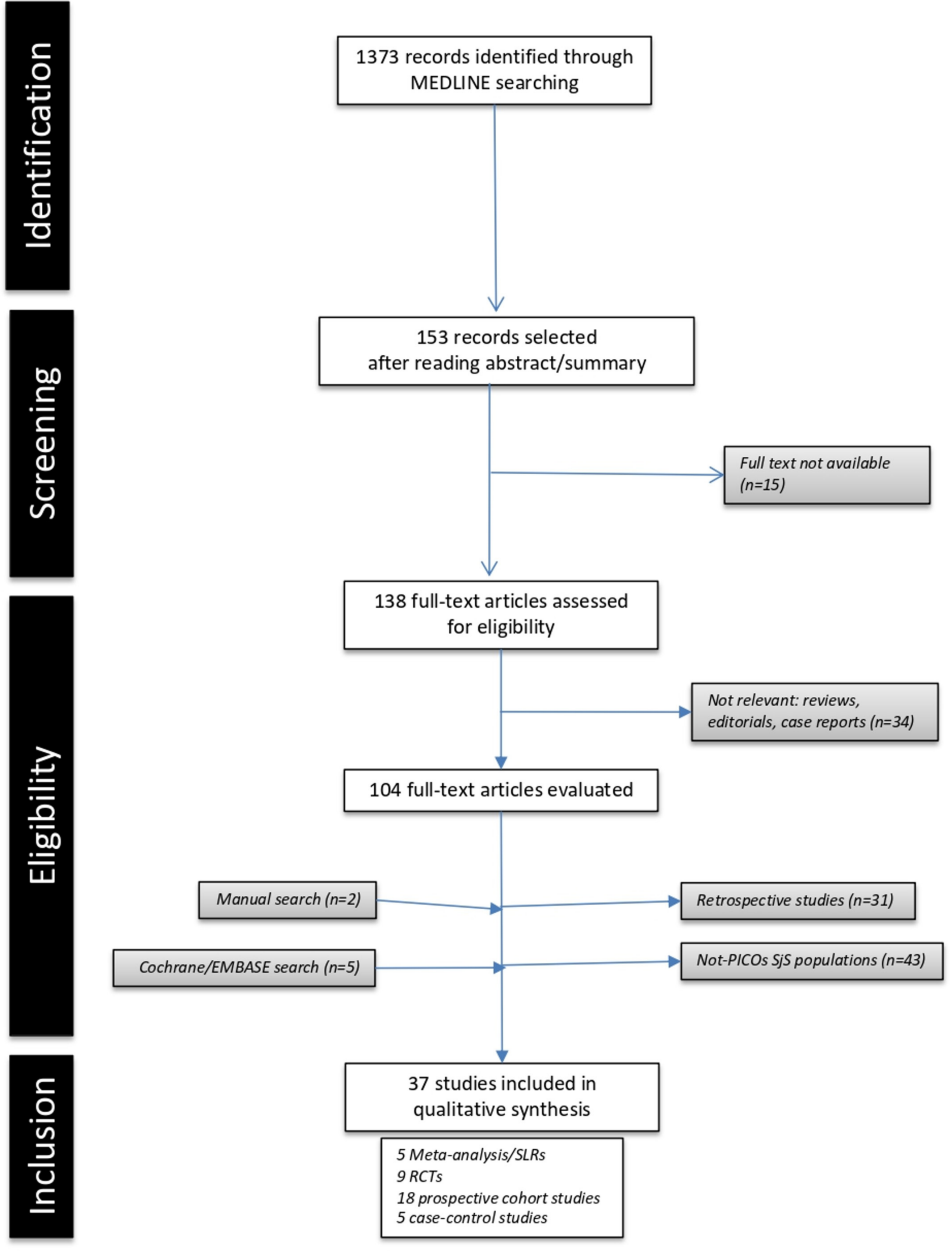
Flow chart of the systematic literature review. PICOs, Population, Intervention, Comparison, Outcomes and Study design; RCTs, randomised controlled trials; SjS, Sjögren’s syndrome; SLRs, systematic literature reviews.

A MEDLINE SLR was carried out by PB-Z and SR using the MeSH term ‘Sjögren’s syndrome’ combined with each therapeutic intervention proposed by the Task Force (see ‘Intervention’ section of the Population, Intervention, Comparison, Outcomes and Study design (PICOS) strategy) with the following restrictions: date (1 January 1986 to 31 December 2017), studies (humans) and age (adults). Additional databases, such as EMBASE and Cochrane Central Library, were also checked. The SLR strategy followed prespecific PICOS definitions agreed by the Steering Committee members: (a) Population: in order to collect evidence from an aetiopathogenically homogeneous population, data was retrieved from studies including adult primary SjS patients fulfilling the 2002 criteria (stated in the manuscript as ‘primary-2002’ patients) or the 2016 ACR/EULAR criteria^8 9^; (b) Intervention: using the data from a 2010 SLR as a starting point,^6 10^ interventions were classified as topical or systemic medications; (c) Comparison: therapeutic interventions were compared with placebo (PLA) or other therapeutic interventions; (d) Outcomes: eligible studies had to contain sufficient, clear information on the effect of the therapeutic intervention (efficacy) and on the safety profile; (e) Study design: we included randomised controlled trials (RCTs), cohort studies (prospective non-PLA-controlled, non-randomised studies and those with quasi-experimental designs), case-control studies (comparing therapeutic options) and meta-analyses, according to the definitions proposed by the Oxford Centre for Evidence-Based Medicine (CEBM),^11^ while case series (descriptive/retrospective therapeutic studies) were considered in the absence of other studies; narrative reviews, experimental animal studies, duplicate publications and isolated case reports were excluded. In the absence of evidence on the target population, extrapolation of results from studies including SjS populations that differed from the definition in the PICOS strategy was allowed. Figure 1 summarises the SLR results. Summary-of-finding tables were generated for RCTs (table 1),^10 12–19^ prospective cohort studies (table 2)^20–37^ and case-control studies (table 3).^38–42^ For RCTs, the risk of bias (RoB) was assessed using the Cochrane RoB assessment tool (Cochrane Handbook for Systematic Reviews of Interventions V.5.1.0 March 2011 (available from: http://handbook.cochrane.org/)), and for uncontrolled studies, we used the Strengthening the Reporting of Observational Studies in Epidemiology (STROBE) statement checklist. The few RCTs available for each therapeutic intervention, together with the heterogeneity in the methodology of the studies included, such as differing participant characteristics, comparative interventions, the small size of the populations studied and the differences in follow-up intervals and outcomes measured, make it impossible to pool data in a meta-analysis.

**Table 1:** Summary-of-findings table generated for RCTs in primary-2002 patients with Sjögren syndrome

Author (year)	No. patients	RoB	Arms (patients)	Primary outcome (drug vs PLA arms, p value)	Secondary outcomes (p value)	SAEs (% of patients in each arm)	Infections	Deaths
Mariette *et al* (2004)^12^	103	Low	INF (n=54) PLA (n=49)	Improvement 30% joint pain, fatigue, dryness VAS at 22 w (20.4% vs 16.7%, p=0.62)	Gammaglobulin (0.05), IgM (0.001) Salivary flow rate, mL/min (p=0.24), Schirmer test (p=0.75), swollen joint count (p=0.75), tender joint count (p=0.97), ESR (p=0.97), CRP (p=0.96), IgA (p=0.56). Focus score (p=0.46).	INF (n=6) vs PLA (n=1)	Not detailed	None
Dass *et al* (2008)^13^	17	Unclear comparative presentation of results	Rituximab (n=8), PLA (n=9)	Improvement >20% VAS fatigue at 6 months (87% vs 56%, p=0.36)	SF-36: social functioning (0.01) NS: Immunoglobulin levels, titres or positivity for other antibodies, glandular manifestations of pSS, Schirmer-I test score, uSF rate.	RTX (n=2) vs PLA (n=0)	Not detailed	None
Meijer *et al* (2010)^10^	30	Unclear (arms not balanced for baseline SF)	Rituximab (n=20) PLA (n=10)	Improvement of SWSF rate at 48 weeks (p>0.05)	VAS oral dryness (p<0.05), VAS ocular dryness (p<0.05)	Not classified as SAEs	RTX 12 in 11 patients vs PLA 7 in 4 patients	None
Norheim *et al* (2012)^14^	26	Moderate (27% men, required 2 phases separated 2 years)	Anakinra (n=13), PLA (n=13)	Group-wise comparison of fatigue scores at week 4 (p=0.19)	Improvement >50% fatigue VAS (0.03) NS W48: Lacrimal gland function, Schirmer’s test, mm/5 min, tear breakup time, seconds 3, 2; SF-36 total score, MFI, general fatigue. W 24Raynaud’s phenomenon (p=0.057), tendomyalgia (p=0.074), arthralgia (p=0.058)	AKR (n=1) vs PLA (n=0)	None	None
Devauchelle-Pensec *et al* (2014)^15^	122	Low	Rituximab 1 g/15 days (n=63), PLA (n=57)	30 mm or greater improvement at week 24 on at least 2 of 4 VAS scores—dryness, fatigue, pain, global (23% vs 22%, p=0.91)	IgG (0.003), IgA (0.026), IgM (0.004) ESSDAI score (p=0.60), systemic signs (p=0.089), salivary flow rate, mL/min (p=0.80), Schirmer test result, mm (p=0.054), ESR, mm/h (p=0.84), serum CRP level, mg/L (p=0.95), C4 complement level, g/L (p=0.32). B2-Microglobulin level, g/L (p=0.35), SF-36 score: PCS (p=0.36), MCS (p=0.35).	RTX 20.6% vs PLA 14%	RTX 52.4% vs PLA 52.6%	None
Gottenberg *et al* (2014)^16^	120	Low	HCQ 400 mg/day (n=56) vs PLA (n=64)	30% or greater reduction at week 24 in 2 of 3 VAS scores—dryness, fatigue, pain (17.6% vs 17.3%, p=0.96)	ESR (<0.001), CRP (0.03), IgM (0.004) ESSPRI (p=0.87), ESSDAI (p=0.63), cardinal signs (pain, fatigue, dryness) evaluated by practitioner (p=0.76), systemic signs evaluated by practitioner (p=0.49), SF-36, physical health component (p=0.85), SF-36, mental health component (p=0.23), HAD-anxiety (p=0.54), HAD-depression (p=0.26), Schirmer test (p=0.42), uSF, mL/min (p=0.45), serum IgG, g/L (p=0.13), serum IgA, g/L (p=0.85).	HCQ 3.6% vs PLA 4.7%	ND	Pneumococcal meningitis (PLA group)
Ho Yoon *et al* (2016)^17^	26	High (primary outcome undefined)	HCQ 300 mg/day (n=11), PLA (n=15)	Not defined	Fluorescein staining score (p=0.524), Schirmer test score (p=0.958), OSDI (p=0.292), TBUT (p=0.746), ESR (p=0.620), serum IL-6 (p=0.991), serum and tear BAFF (NA), Th17 cells (p=0.566).	Not classified as SAEs	None	None
Bowman *et al* (2017)^19^	133	Low	Rituximab 1 g/15 days (n=67), PLA (n=66)	Reduction ≥30% at week 48 of either fatigue or oral VAS dryness (39.3% vs 36.8%, p=0.76)	uSF (0.0015) ESSPRI (p=0.1087), ESSDAI scores (p=0.0721), mean lacrimal flow (p=0.3698), SF-36 physical component (p=0.5246), SF-36 mental component (p=0.9495). PROFADSSI domains (p>0.05)	RTX n=9 vs PLA n=9	RTX n=2 vs PLA n=2	None
St Clair *et al* (2018)^18^	52	Unclear (study enrolment was terminated early because of expiration of study drug)	BAM (n=33), PLA (n=19)	Change in the SWSF rate at week 24 (+0.07 vs −0.01, p=0.33)	Schirmer test right eye (0.036) Unstimulated WSF (p=0.881), ESSDAI (p=0.104), physician global assessment (p=0.646), subject global assessment (p=0.587), overall dryness (p=0.744), fatigue (p=0.737), joint pain (p=0.797), Schirmer I test (mm)2 Left eye (p=0.662) Total ocular staining score (p=0.603), SF-36: Physical aggregate score (p=0.163), Mental aggregate score (p=0.885). BAFF (pg/mL) (p=0.523), LIGHT (pg/mL) (p=0.840), IP-10 (pg/mL) (p=0.907)	BAM 15% vs PLA 5%	BAM 24.2% vs PLA 15.8%	None

AKR, anakinra; BAFF, B-Cell Activating Factor; BAM, baminercept ; CRP, C-reactive protein; ESR, erythrocyte sedimentation rate; ESSDAI, EULAR Sjögren's syndrome disease activity index; ESSPRI, EULAR Sjogren's Syndrome Patient Reported Index; HAD, Hospital Anxiety and Depression Scale; HCQ, hydroxychloroquine; IL-6, interleukin 6; INF, infliximab; MCS, Mental Health Composite Score; MFI, Multidimensional Fatigue Inventory; ND, not detailed; NS, not significant; OSDI, Ocular Surface Disease Index; PCS, Physical Health Composite Score; PLA, placebo; PRO-FAD-SSI, Profile of Fatigue and Discomfort-Sicca Symptoms Inventory; pSS, primary Sjögren syndrome; RCT, randomised controlled trial; RoB, risk of bias; RTX, rituximab; SAEs, serious adverse events; SF-36, Short Form-36 Health Survey; SWSF, stimulated whole salivary flow; TBUT, Tear breakup time; uSF, unstimulated salivary flow; VAS, visual analogue scale; VAS, visual analogue scala; W, week; WSF, whole salivary flow.

**Table 2:** Summary-of-findings table generated for prospective studies in primary-2002 patients with Sjögren syndrome

Author (year)	Patients	Design (duration)	Intervention, dose (patients)	Comparison (patients)	Efficacy parameters (p<0.05)		Safety profile
					Significant associations (p<0.05)	Non-significant associations (p>0.05)	
Kedor *et al* (2016)^26^	30	Prospective (16 w)	Oral cyclosporine A, approx 2 mg/kg/day (n=30)	None	Tender joint count (0.001), swollen joint count (<0.001), DAS28 (<0.001), ESSDAI (<0.001), gammaglobulin (0.009), anti-La (0.048)	Patient’s disease activity (p=0.249), pain (p=0.094), fatigue (p=0.350),SF-36 total (p=0.259), HAQ-DI (p=0.372), CRP mean (p=0.780), ESR mean (p=0.268), IgG mean (p=0.360), Schirmer’s test (p=0.820), Saxon’s test (p=0.925), anti-Ro (SSA) 60 kDa (p=0.786), anti-Ro (SSA) 52 kDa (p=0.400), RF (p=0.099)	All had experienced at least one adverse event (AE): gastrointestinal (70%), muscle craps (67%), nervous system (53%), skin (53%); infections (30%) of mild or moderate severity occurred 13 times in 10 patients; drop-out 6/28 (21%)
Egrilmez *et al* (2011)^20^	22	Prospective (12 m)	Plug (n=22)	None	Schirmer test (0.006), BUT (<0.001)	Visual acuity levels (p=0.608), lissamine green staining scores (p=0.958)	Pyogenic granuloma (n=1)
Aragona *et al* (2006)^21^	15	Prospective	Pilocarpine	NA	Dry mouth (<0.001)	VARS for systemic symptoms (NS): skin dryness, vagina dryness.	Sweating in 6 (40%), chill in 3 (20%), nausea in 2 (13%), oversalivation in 2 (13%), gastritis in 1 (7%)
		(2 m)	5 mg/6 hours (progress increase of dose)		Ocular burning, foreign body (<0.02)	VARS for ocular symptoms (NS): itching, mucus secretion, photophobia, hyperaemia, tearing.	
						Ocular tests results (NS): corneal fluorescein stain, Schirmer’s I, test basal secretion test	
Yamada *et al* (2007)^30^	13	Prospective	Cevimeline 30 mg	No	No information about overall efficacy	Groups according to positive or negative findings of: sialography: age (p=0.700), labial minor salivary gland biopsy: age (p=0.623), pretreatment WSS (p=0.806), post-WSS (p=0.073) anti-Ro/SSA antibodies: age (p=0.446), pretreatment WSS (p=0.268), post-WSS (p=0.165), increment rate (p=0.683) anti-La/SSB: age (p=0.561), pretreatment WSS (p=0.914), post-WSS (p=0.116), increment rate (p=0.018) Disease duration (months): age (p=0.917), pretreatment WSS (p=0.934), post-WSS (p=0.950), increment rate (p=1.000)	No serious adverse effects
		(4 w)	One time daily (first 2 w)		Higher increase of WWS in patients with:		
			Two times (next 2 w)		Negative sialography (0.042), negative La (0.018) and negative bx (0.002)		
Yavuz *et al* (2011)^31^	32	Prospective	HCQ 6.5 mg/kg/day (>2 years)	No	Symptom severity score (<0.001)	OSDI (NS), Schirmer’s test (mm) NS, Schirmer’s test with anaesthesia (mm) NS, average tear drop/day NS, NEI-VFQ-25 questionnaire (NS)	Not detailed
		(12 w)			Tear BUT (0.001) corneal fluorescein (0.01)		
					Oxford score (0.003)		
Cankaya *et al* (2010)^32^	30	Prospective	HCQ 400 mg/day	No	Mean uSFR (<0.05)	Dry mouth (p=0.292), burning oral mucosa (p=0.11), difficulty in mastication (p=0.969)	Not detailed
		(30 w)					
van Woerkom *et al* (2007)^27^	15	Prospective	Leflunomide 20 mg/24 hours	No	MFI (0.034)	VAS general health (p=0.529), VAS dry eyes (p=0.361), VAS sandy feeling (p=0.343), VAS dry mouth (p=0.098), VAS sleep disturbance due to dryness (p=0.484), Zung depression score 37 (p=0.726), RAND (SF-36) mental component (p=0.790), ESR (p=0.200), CRP (p=0.453), Schirmer test (p=0.138), sialometry (p=0.632)	All 15 patients suffered AEs; not classified as SAEs
		(24 w)			SF-36 physical component (0.026)		Diarrhoea 7, GI discomfort 6, hair loss 7, weight loss >2 kg 5
					Reduced serum IgA (0.023), IgG (0.006) and IgM (0.005)		Headache 5, LE skin lesions 5, anaemia 5, leucop 4, dizziness 4
					Reduced RF levels (0.045)		TAS 3, rashes 4 (different patients of LE rashes)
Willeke *et al* (2007)^28^	11	Prospective	Mycophenolic acid	No	VAS sicca (<0.02)	Schirmer's test (millimetres per 5 min), whole saliva (grams per 5 min), VAS arthralgia, VAS fatigue, Health Assessment Questionnaire score, erythrocyte sedimentation rate (mm/hour), IgG (mg/dL), IgA (mg/dL), anti-SSA antibodies, anti-SSB antibodies. No changes in the 28-swollen/tender joint count or in the number of tender points were observed (data not shown). No significant changes concerning the Raynaud syndrome were observed.	Three withdrawals (one pneumonia)
		(24 w)	Increased dose		Mean AT use (<0.02)		Total AE: 7/11 (63%); not classified as SAEs
			(360 mg to 1440 mg daily)		Reduct gammaglobulins, C3 and C4 levels (<0.02)		GI discomfort=5, herpes=1, common cold=2
					Reduct IgM, RF (<0.05)		
					Increased leucocytes (<0.05)		Dose reduction in 2
					General health, role emotional SF-36 domains (<0.05)		
Zandbelt *et al* (2004)^29^		Prospective	Etanercept 25 mg twice per week	No	CRP (<0.05)	ESR (p=0.058), gammaglobulin (p>0.05), Schirmer-I tests (p>0.05), SL/SM salivary (p>0.05), flow measurements (p>0.05), BUT or rose bengal staining (NS, data not shown). Post-treatment LFS (p=0.101) and IgA% (p=0.621). Raynaud syndrome (NS).	Infectious parotiditis (n=1)
		12 w	(n=15)		General fatigue scale within the MFI (p=0.018)		
					VAS score for perceived disease activity (p=0.045)		
Pijpe *et al* (2005)^33^	15	Prospective (12 w)	Rituximab 375 mg/m2	No	Only in the subset ‘early’: rose bengal, BUT, MFI, SF-36 PF, V, HC (<0.05)	Either group of patients: levels of IgG, IgA, IgM, and 2-microglobulin did not change. Patients with MALT/primary SS: No changes in T-cell subsets All patients (>0.05): whole saliva, stimulated submandibular/sublingual salivary secretion.Schirmer’s test. Patients with MALT/primary SS: rose bengal, BUT, MFI, SF-36 PF, V, HC (>0.05)	Infusion-related (n=2), Herpes zoster (n=1), HACAs: 4/8 of early SS, 0/7 in MALT group, serum sickness (n=3), all HACA+
Devauchelle-Pensec *et al* (2007)^34^	16	Prospective (36 w)	Rituximab 375 mg/m^2^	No	Global VAS (0.03), pain VAS (0.006), fatigue VAS (0.006), dryness VAS (0.006), tender point count (0.027), tender joint count (0.017), IgA-RF (0.04)	Ocular and oral dryness (p>0.05), swollen joint count (p=0.15), salivary flow rate, mL/min (p=0.86), Schirmer test (p=0.79), anti-SSA (p=0.25). ESR (p=0.6), Latex test (p=0.1), IgA (p=0.7), IgG (p=0.2), IgM (p=0.2)	Infusion-related (n=2), lymphoma (n=1), delayed reactions (n=8), serum sickness (n=4)
St Clair *et al* (2013)^35^	12	Prospective (26 w)	Rituximab 375 mg/m^2^	No	Global VAS physician (0.012) and patient (0.009), VAS tongue dryness (0.007), level of thirst (0.005), oral discomfort (0.02), fatigue (0.042)	Joint pain (p=0.077), unstimulated (p=0.287) or stimulated (p=0.718) whole salivary flow, RF (p=0.109) p≥0.05: Tear production, Schirmer’s test, ocular surface dryness (von Bijsterveld scoring system), SF-36 for physical and mental functioning between week 0 and week 26.	Severe AE reaction to pneumococcal vaccine (n=1); non-severe (n=2), squamous cell carcinoma (+301 d)
Carubbi *et al* (2013)^36^	41	Case control (120 w)	Rituximab 1 g/15 d (n=22)	DMARD treatment (n=19)	ESSDAI reduction RTX vs DMARD (<0.05)	Unstimulated salivary flow and the Schirmer’s I test were not affected in the DMARD treatment group.	No adverse events
					Global VAS (<0.05), fatigue VAS (<0.01), dryness VAS (<0.01), physician VAS (<0.05), uSF (<0.01), Schirmer (<0.05)	p>0.05: IgG, ANA, RF, anti-Ro/SSA and anti-La/SSB antibodies	No withdrawals
Mariette *et al* (2015)^57^	30	Prospective (28 w)	Belimumab 10 mg/kg	No	Dryness VAS (0.0021), ESSPRI (0.0174), ESSDAI (0.0015)	Unstimulated whole salivary flow (p=0.27) or Schirmer’s test (p=0.51), even in SF-36 physical health and mental health component (p=0.71) The focus score of the lymphoid labial salivary gland (LSG) infiltrate (p=0.57). Mean baseline BAFF level (p=0.57) Decrease of three points or more of ESSDAI (p=0.44).	Pneumococcal meningitis (n=1), breast cancer (n=1), scleroderma (n=1), pneumonia (n=1), headache (n=9), sinusitis (n=1), neutropenia (n=5), Rhinitis/pharyngitis (n=7), oral aphtosis (n=1), bronchitis (n=1), Herpes labialis (n=1), urinary tract infection (n=2), gastroenteritis/diarrhoea (n=2)
					ESSDAI glandular (0.0078), biologic (0.0078), articular (0.0313)		
De Vita *et al* (2015)^22^	19	Prospective extension (52 w)	Belimumab 10 mg/kg	No	Physician VAS (0.04), RF (0.048), IgM (<0.01) Glandular domain (p=0.0078) Articular domain (p=0.0313) Biologic domain (p=0.0078)	VAS dryness score (p=1.0), VAS fatigue (p=0.14) VAS pain (p=0.71), biologic improvement (p=1.0) at W28 and W52. VAS score of disease systemic activity by the physician at W28 (p=0.65), p>0.05: SF-36 physical health, mental health component uSFR (p=0.6), Schirmer’s I test (p=0.3) Focus score of labial salivary gland biopsy (p=0.9) Lymphadenopathy domain (p=0.0625)	Rhinopharingitis (n=2), headache at the end of the infusion (n=1), gastroenteritis (n=1), mild transient neutropenia (n=2), urinary tract infection (n=1), pneumonia (n=1), vaginal fungal infection (n=1), non-complicated cutaneous infection (n=1)
Steinfled *et al* (2006)	16	Prospective (18 w)	Epratuzumab 360 mg/m^2^	No	VAS fatigue (<0.05), patient assessment (<0.05), physician assessment (<0.05), tender joints (<0.05)	p>0.05: CRP; ESR; Ig, pain, changes from baseline in T cells	Severe infusion related (n=1) (discontinued), sinusitis (n=1), transient ischaemic attack with secondary seizure (n=1), moderate grade-3 acute infusion reaction (n=1), discontinued at third infusion, dental abscess (n=1), osteoporotic fracture (n=1), mild infusion related (n=2), headache, paresthesia (n=3), fever, palpitation, bone pain, carpal tunnel syndrome, diarrhoea, and dyspepsia (ND)
Meiners *et al* (2014)^24^	15	Prospective (48 w)	Abatacept 10 mg/kg	No	ESSDAI (<0.05), ESSPRI (<0.05), Patient’s GDA (<0.05), Physician’s GDA (<0.05), RF (klU/L) (<0.05), IgG (g/L) (<0.05)	ESSDAI at W48 from baseline (p=0.137) ESSPRI post-treatment (p=0.151) Unstimulated whole saliva, parotid flow rate and lacrimal gland function, patient’s GDA, parotid saliva, stimulated (mL/min), Schirmer (mm/5 min): NS.	No SAEs occurred, and no patients withdrew from the study due to AEs.
							Mild infusion reaction (n=1); mild acute AEs -dizziness, hypotension- (17 events in 6 patients)
							18 self-reported infections (18 infections in 10 patients), the most common being upper respiratory tract infections. No infection required hospitalisation.
Adler *et al* (2013)^25^	11	Prospective (108 w)	Abatacept 500–750 mg	No	Numbers of lymphocytic foci decreased (0.041), numbers of local FoxP3, T cells decreased (0.037), peripheral blood, B cells increased (0.038), expansion of the naive B cell pool (0.034)	Histology (NS): Lymphocytic foci/mm2, CD20 B cells, CD3 T cells, mm2, CD20 B cells, CD3 T cells, CD4 T cells, CD8 T cells.	No serious adverse events, no infusion reactions
					Total lymphocytes increase (0.044) and for CD4 cells (0.009)	Serum (NS): IgG, g/L	Transient increase in liver enzymes (concomitant rifampin) (n=1)
					Gamma globulins decreased (0.005)	Peripheral blood cells (NS): lymphocytes, CD3 T cells, CD4 T cells, CD8 T cells, memory B cells, switched memory B cells, non-switched memory B cells	Diverticulitis (n=1)
					Saliva production increased (0.029)		Lupus-like skin lesions (n=1)

AEs, serious adverse events; ANA, antinuclear antibody; AT, artificial tears; BAFF, B-Cell Activating Factor; BUT, tear breakup time; bx, biopsy; CRP, C-reactive protein; DAS, disease activity score; DMARD, Disease-modifying anti-rheumatic drug; ESR, erythrocyte sedimentation rate; ESSDAI, EULAR Sjögren's syndrome disease activity index; ESSPRI, EULAR Sjogren's Syndrome Patient Reported Index; GDA, global disease activity; GI, gastrointestinal; HACA, human antichimeric antibodies; HAQ-DI, Health assessment questionnaire disability index; HC, health change; HCQ, hydroxychloroquine; LE, lupus erythematosus; LFS, lymphocyte focus score; m, month; MALT, mucosa-associated lymphoid tissue–type lymphom; MFI, Multidimensional Fatigue Inventory; ND, not detailed; NEI-VFQ-25, National Eye Institute-Visual Function Questionnaire-25; NS, not significant; OSDI, Ocular Surface Disease Index; PF, physical functioning; RF, rheumatoid factor; RTX, rituximab; SAEs, serious adverse events; SF-36, Short Form-36 Health Survey; SL/SM salivary, sublingual/submandibular gland; TAS, taste; uSFR, unstimulated salivary flow rate; V, vitality; VARS, Visual analogue rating scales; VAS, visual analogue scale; w, week; WSS, Whole stimulated sialometry.

**Table 3:** Summary-of-findings table generated for case-control studies in primary-2002 patients with Sjögren syndrome

Author (year)	Patients	Design (duration)	Intervention, dose (patients)	Comparison (patients)	Differences between-groups (p value)	Differences within-groups (p value)	Safety profile
Alpöz *et al* (2008)^38^	29	Case control	Xialine	Water	Relief xerostomia complaints (0.06)	p values not detailed	Not detailed
		(2 w)			VAS improvement for Xialine group in mastication (0.06), swallowing (0.027), daily liquid consumption (0.019), mouth burning (0.025), the need to sip liquids to aid swallowing (0.023), difficulty in speaking (0.004)		
					VAS satisfaction better for Xialine (0.011)		
			VAS satisfaction better for Xialine (0.011)		No differences for VAS burning tongue (0.925), diminished taste (0.527), waking up at night to sip water (0.066)		
Qiu *et al* (2013)^39^	40	Case control (nd)	Plug (n=21)	Artificial tears (AT) (n=19)	OSDI score, BUT, Schirmer I, corneal staining score (p>0.05)	Plug group: better OSDI score, BUT, Schirmer I, corneal staining score (p<0.001)	Not detailed
						AT group: better OSDI score, BUT, Schirmer I, corneal staining score (p<0.001)	
Lin *et al* (2015)^40^	40	Case control	0.1% fluorometholone (FML) (n=20)	Topical cyclosporine A (n=20)	CFS score (>0.05), OSDI score (>0.05), Schirmer (>0.05), conjunctival goblet cell density (p<0.001)	FML group: better CFS score (<0.001), BUT (<0.001), OSDI score (<0.001), Schirmer (>0.05), conjunctival goblet cell density (ns), conjunctival congestion at week 4 (p=0.035)	No serious or severe adverse effects occurred
		(8 w)			Mean BUT longer in FML group (0.04)	CyA group: better CFS score (<0.001), BUT (<0.001), OSDI score (<0.001), Schirmer (>0.05), conjunctival goblet cell density (ns)	Moderate/severe transient burning sensation (CsA 31.25%, FML 0%)
							Less severe conjunctival congestion in FML group at week 4 compared with CsA group (p=0.035)
							Mean IOP +0.4 mm Hg FML vs −1.15 mm Hg CsA (p=0.389)
Li J *et al* (2015)^41^	37	Comparative	Autologous serum (AS) (n=18)	Bandage contact lens (BCL) (n=19)	BUT (>0.05), corneal staining (>0.05), Schirmer (>0.05), BCVA (>0.05)	AS group: BUT (0.001), corneal staining (0.001), Schirmer (>0.05), BCVA (>0.05)	No adverse events
		(6 w)					
					OSDI: 47.1 AS vs 31 BCL (<0.01)	BCL group: BUT (<0.001), corneal staining (<0.001), Schirmer (>0.05), BCVA (0.003)	
							
Noaiseh *et al* (2014)^42^	118	Case control	Pilocarpine first line (n=59)	Cevimeline first line (n=59)	Failure rates among first-time users: Cevimeline vs pilocarpine 27% vs 47% (p=0.02)	ANA (+) was associated with failure:(59% vs 38%) (p=0.03)	Pilocarpine first line: 28 patients (47%) discontinued treatment due to AE.
		(2.8 y)	Pilocarpine second line (n=13)	Cevimeline second line (n=32)	Failure rates among all users: Cevimeline vs pilocarpine 32% vs 61% (p<0.001).		Sweating (n=15), nausea, dyspepsia or vomiting (n=6), flushing/hot flashes (n=3), paresthesias (n=1), myalgias (n=1), headaches (n=1) and rash (n=1).
					Cevimeline (first-time users) had lower failure rates due to AE vs pilocarpine (p=0.02)		11 patients (19%) discontinued therapy due to lack of efficacy
					Previously failed one secretagogue were less likely to discontinue treatment with the other agent, 52% of first-time users vs 27% of second-time users (p=0.004).		Cevimeline first line: 16 patients (27%) discontinued due to AE:
							Sweating (n=8), nausea, dyspepsia and vomiting (n=5), flushing/hot flashes (n=1), headaches (n=1) and breast swelling (n=1)
							6 patients (10%) due to lack of efficacy
							Pilocarpine second line: 3 patients (23%) developed AE requiring discontinuation
							Sweating (n=1), dyspepsia (1) and flushing/hot flashes (1)
							Two patients stopped treatment due to lack of efficacy
							Cevimeline second line: 7 (22%) developed AE requiring discontinuation
							Sweating (n=2), dyspepsia (1), flushing flushing/ hot flashes (1), diarrhoea (1), parotid swelling (1) and postnasal drip (1)
							None stopped treatment due to lack of efficacy
							Severe sweating more frequently in pilocarpine (25%) than cevimeline (11%) users (p=0.02)

ANA, antinuclear antibody; AT, artificial tears; BCVA, best-corrected visual acuity; BUT, tear breakup time; CFS, Corneal Fluorescein Staining; CyA, cyclosporine A; CyA, cyclosporine A; FML, fluorometholone; IOP, intraocular pressure; OSDI, Ocular Surface Disease Index; VAS, visual analogue scale; w, week.

## Results

### Oral topical therapies

#### Saliva substitutes

We identified five studies that evaluated gels/saliva substitutes in SjS patients, of which only one was carried in primary-2002 patients^38^: Alpöz *et al* found that Xialine (a saliva substitute containing polysaccharide xanthan gum plus sodium fluoride) and plain water plus diluted tea (serving as PLA) were equally effective in most VAS scoring for specific oral symptoms, with the only between-group differences being an increased preference for Xialine at the end of the study (p=0.011). A Cochrane SLR evaluated the effectiveness of topical treatments for any-cause dry mouth (including SjS) in parallel and crossover RCTs using lozenges, sprays, mouth rinses, gels, oils, chewing gum and/or toothpastes and found no strong evidence supporting any one specific topical therapy as more effective in treating dry mouth.^43^

#### Interferon alpha

Three studies have evaluated the use of interferon alpha per the oromucosal route in SjS patients fulfilling the 1993 criteria, including a large RCT of nearly 500 patients that found significant improvement only in unstimulated salivary flow (uSF), with a higher percentage of gastrointestinal adverse events in comparison with PLA.^3^

### Ocular topical therapies

#### Artificial tear drops

Seven studies testing artificial tears (ATs) in patients with SjS were identified, all of which found significant improvements with respect to baseline in both VAS ocular dryness and diagnostic tests (except in one study) with no reported side effects.^3^ Only one study,^39^ comparing the use of AT with plug insertion, was carried out in primary-2002 patients: no significant between-group differences were reported and, after 8 weeks of treatment, patients treated with AT showed significant improvement in all ocular diagnostic tests performed (p<0.001). A recent Cochrane review of AT drops for dry eye syndrome concluded that ATs are safe and effective.^44^

#### Non-steroidal anti-inflammatory drugs/GC-based tear drops

Evidence is overwhelmingly limited to studies including patients with associated SjS or non-2002 SjS patients. Only one study^40^ was carried out in primary-2002 patients, and this compared topical 0.1% fluorometholone (FML) with topical CyA: although no significant differences were detected between groups for the main efficacy parameters (except for tear breakup time (BUT), with better results in the FML group), although patients treated with topical 0.1% FML showed significant improvements with respect to baseline in the Corneal Fluorescein Staining score (p<0.001), BUT (p<0.001) and Ocular Surface Disease Index (p<0.001) after 8 weeks of therapy, but not for the Schirmer test; no serious side effects were reported: the mean intraocular pressure change at 8 weeks was +0.4 mm Hg in the FML group versus −1.15 mm Hg in the CyA group (p=0.389).

#### Cyclosporine-based tear drops

In December 2002, an ophthalmic formulation containing 0.05% CyA was approved by the US FDA to treat dry eye disease at a recommended two times per day dose, based on the results of two RCTs that included 1039 patients with keratoconjunctivitis sicca (SjS patients were included in varying proportions).^3^ Since then, a summary of the results reported until now shows that most studies only demonstrated within-group improvements, and those that reported significant between-group differences (drug and vehicle) found improvement in only one to two of the four to eight ocular outcomes evaluated.^45^ In addition, a study extension trial found no additional improvement in subjective and objective measurements after 6 months of therapy.^46^ With respect to adverse events, the largest RCT^47^ found that most events were mild-to-moderate and transient, with a significantly higher percentage of burning eye in comparison with PLA (15% vs 6% in PLA), and only 2% of patients discontinued because of burning and stinging.^3^

There are no specific RCTs in primary-2002 patients, with only one case-control study, the above-mentioned study by Lin and Gong,^40^ which showed no significant differences with topical 0.1% FML and with a higher frequency of moderate-to-severe transient burning sensation in patients receiving CyA.

#### Tacrolimus-based tear drops

A recent small RCT using 0.03% tacrolimus tear drops in 24 SjS 2002 patients found significant improvements in the Schirmer test and corneal staining (fluorescein, rose Bengal) after 3 months of therapy in comparison with drops that included only the vehicle. Nearly 80% of those receiving tacrolimus experienced a burning sensation after instillation.^48^

#### Serum tear drops

Autologous serum (AS) has been tested in six small (<30 patients) uncontrolled studies in SjS patients and has shown inconsistent benefits (improvement in some but not all ocular tests performed).^49^ Only one study was carried out in primary-2002 patients, which showed a significant improvement with respect to baseline in three out of five ocular outcomes.^41^ A recent Cochrane review of AS for dry eye syndrome^49^ confirmed inconsistency in the possible benefits of AS for both symptoms and objective measures, with no evidence of an effect after 2 weeks of treatment.

#### Insertion of lachrymal plugs

Of the nine studies identified in SjS patients, only two were carried out in p2002-SjS patients. Qiu *et al*^39^ compared the insertion of plugs with ATs in 40 patients: both treatments improved all ocular outcomes (subjective and objective) without statistically significant between-group differences. In a prospective study, Egrilmez *et al*^20^ reported improvement in two out of four ocular tests with respect to baseline 12 months after inserting plugs. A recent Cochrane SLR reviewed the use of plugs for dry eye syndrome^50^ and found that the evidence was very limited and improvements in symptoms and ocular tests were inconclusive.

#### Diquafosol

In 2004, an ophthalmic formulation of diquafosol (an agonist of the purinergic P2Y receptor) was tested by Tauber *et al*^51^ in an RCT including 527 patients with dry eye (only 76 had SjS), which reported statistically significant differences between groups in only one of the two defined primary outcomes, with no further studies being reported, making it impossible to recommend their use.

### Oral muscarinic agonists

Two muscarinic agonists (pilocarpine and cevimeline) were licensed by the FDA in 1998 and 2000, respectively, for the treatment of oral dryness in SjS patients; these agents stimulate the M1 and M3 receptors present on salivary glands, leading to increased secretory function.

#### Pilocarpine

The two pivotal RCTs included 629 SjS patients (fulfilling the 1993 criteria and including both primary and associated cases) and found significant improvements in oral dryness VAS and salivary flow rates at doses of 5 and 7.5 mg/6 hours in comparison with the PLA arm.^3^ The RCTs showed a high frequency of adverse events, including sweating (43%), increased urinary frequency (10%) and flushing (10%). In a dose-escalating RCT, nearly a quarter of patients reduced from 7.5 to 5 mg/6 hours in a second 6-week period of therapy. Only two studies have been carried out in primary-2002 patients, and only one assessed efficacy. In a prospective study, Aragona *et al*^21^ reported a significant improvement with respect to baseline in oral dryness VAS (p<0.001) after 2 months of therapy with pilocarpine 5 mg/6 hours.

#### Cevimeline

Three RCTs including 332 SjS patients (fulfilling the 1993/Japanese criteria, both primary and associated cases) tested the use of cevimeline using dosages ranging between 15 and 60 mg/8 hours. The best results were achieved with a dose of 30 mg/8 hours, including significant improvements in dry mouth and salivary flow rates, with a significantly higher frequency of nausea (relative risk 1.68) and sweating (relative risk 2.16) in comparison with PLA.^3^ There is only one study carried out in primary-2002 patients, but there was no information detailed about overall efficacy and safety.^30^ Only one study has compared cevimeline with pilocarpine but only assessed the safety profile. Noaiseh *et al*^42^ retrospectively analysed 118 primary-2002 patients and found a lower failure rate of cevimeline both in first-time (27% vs 47%, p=0.02) and all (32% vs 61%, p<0.001) users in comparison with pilocarpine. Severe sweating was the main reason for therapy cessation and occurred more frequently in pilocarpine users (25% vs 11%, p=0.02).

### Hydroxychloroquine

We identified 12 studies that assessed hydroxychloroquine in SjS patients, with only 4 (2 prospective and 2 RCTs) carried out in primary-2002 patients. Yavuz *et al*^31^ prospectively enrolled 32 patients treated with hydroxychloroquine for at least 2 years (no data on mean length or cumulative dose) and reported, in a further 12-week control study, a significant improvement in four out of eight ocular outcomes with respect to baseline, with no information on concomitant ocular therapies. Çankaya *et al*^32^ prospectively evaluated 30 women who started on 400 mg/day of hydroxychloroquine and reported a significant improvement in uSF rate (0.212 vs 0.162 baseline, p<0.05) but not stimulated salivary flow rate at 30 weeks, with improvement in only two of the five subjective oral VAS scores. Yoon *et al*^17^ carried out a small RCT in 26 patients and found no significant differences in dry eye in comparison with PLA, but with no definition of the primary outcome.^17^ The pivotal RCT was carried out by Gottenberg *et al*^16^ in 120 patients with primary-2002 SjS who were randomised to receive 400 mg/day of hydroxychloroquine (n=56) or PLA (n=64): the primary outcome was defined as a ≥30% reduction in two out of three VAS scores—dryness, fatigue, pain—without significant PLA differences at week 24 (17.6% vs 17.3%, p=0.96). For one of the secondary outcomes, hydroxychloroquine was associated with a statistical trend to improved pain (p values between 0.06 and 0.10 at 12, 24 and 48 weeks) although it was not superior to PLA for articular involvement; with respect to fatigue, no statistical differences were found.^16^ No cases of retinal toxicity or severe adverse events were reported in any of these studies.

### Oral GCs

The frequent use of GCs in clinical practice in primary SjS patients is not supported by reliable scientific evidence. In the absence of controlled studies in 2002 patients, we briefly analysed the data in other SjS populations.^3^ The first study was a very small RCT (eight patients per arm) comparing prednisone 30 mg/day with piroxicam 20 mg/day and PLA, which found significant differences in subjective symptoms but not in objective tests.^52^ However, a prospective study of 60 patients followed for a mean of nearly 4 years found that corticosteroids did not influence the progressive decrease in salivary flow rates.^53^ No controlled studies are published in primary-2002 patients, and only retrospective studies have reported the use of GCs for systemic disease,^54^ with a high rate of GC-related adverse events, including increased appetite and weight gain,^52^ a two-fold higher frequency of diabetes mellitus^55 56^ and Cushing in up to 80% of patients.^56^

### Immunosuppressive agents

Seven studies have tested immunosuppressive agents in SjS patients (two studies using leflunomide and cyclophosphamide, respectively, and one study each for azathioprine, methotrexate and mycophenolate), of which only two were carried out in primary-2002 patients. van Woerkom *et al*^27^ carried out a prospective study using 20 mg/day of leflunomide, which showed significant improvement in 5 out of 16 efficacy parameters tested after 24 weeks of therapy (2 were components of quality of life (QoL) questionnaires and 3 analytical parameters); all 15 patients experienced adverse events (between 4 and 8 per patient), principally gastrointestinal (67%), cytopenia (47%) and lupus-like cutaneous lesions (33%). Willeke *et al*^28^ tested the use of micophenolic acid 1440 mg/day, reporting significant improvement in 8 out of 16 efficacy parameters after 24 weeks of therapy, including VAS for sicca features (p<0.02) and mean AT use (p<0.02) (other parameters that improved included components of QoL questionnaires and analytical parameters); adverse events were reported in 72% of patients.

### Biological therapies

Of the 34 studies in which biological agents have been tested in SjS patients, we identified 6 RCTs (using infliximab (INF), anakinra and rituximab) and 10 prospective cohort studies (using etanercept, abatacept, epratuzumab, rituximab and belimumab) carried out in primary-2002 patients. A summary of the results of the efficacy parameters is provided in table 4.

**Table 4:** Summary of the outcomes evaluated in RCTs including primary-2002 patients with Sjögren syndrome

Author (year)	Patients	Weeks	Drug, dose	Outcomes							
				Global/ composite	Dryness	Pain	Fatigue	HRQoL	Salivary flows	Ocular tests	ESSDAI
Mariette *et al*^12^ (2004)	103	22	Infliximab, 5 mg/kg	**VAS**	VAS	VAS	VAS		SFR	Schir	
Sankar *et al* (2004)^67^	28	12	Etanercept, 25 mg	**VAS**	VAS				SFR	Liss, Schir	
Dass *et al*^13^ (2008)	17	24	RTX, 1 g/15 days				**VAS**	SF-36*			
Meijer *et al*^10^ (2010)	30	48	RTX, 1 g/15 days		VAS*		MFI	SF-36	**SWS**	Liss, BUT, Schir	
Norheim *et al*^14^ (2012)	26	4	Anakinra, 100 mg/day				**VAS**	BDI			
Devauchelle-Pensec *et al*^15^ (2014)	122	24	RTX, 1 g/15 days	**VAS**	VAS	VAS	VAS	SF-36	SFR	Schir	Mean improvement
Bowman *et al*^19^ (2017)	133	48	RTX, 1 g/15 dys	**VAS**	VAS oral		VAS	ESSPRI, SF-36	UWS*	Lachr flow	Log-transf
St Clair *et al*^18^ (2018)	52	24	Baminercept, 100 mg weekly		VAS	VAS	VAS		**UWS, SWS**	Liss, Schir*	Mean score

Grey cells=primary outcome.

*Statistically significant.

BDI, Beck Depression Inventory; ESSDAI, EULAR Sjögren's syndrome disease activity index; ESSPRI, EULAR Sjogren's Syndrome Patient Reported Index; HRQoL, health-related quality of life; MFI, Multidimensional Fatigue Inventory; RCT, randomised controlled trial; RTX, rituximab; Schir, schirmer; SF-36, Short Form-36 Health Survey; SFR, salivary flow rate; SFR, salivary flow rate; UWS, unstimulated whole saliva; VAS, visual analogue scale.

#### Abatacept

Two small prospective cohort studies have tested abatacept in primary-2002 patients. The first enrolled 15 patients with early active disease who received eight intravenous abatacept infusions^24^ and reported that ESSDAI, EULAR Sjogren’s Syndrome Patient Reported Index (ESSPRI), rheumatoid factor and IgG levels decreased significantly at 48 weeks; fatigue and health-related quality of life parameters improved significantly, while salivary and lacrimal gland function did not change; 6 (40%) patients experienced mild acute adverse events and 10 (67%) self-reported infections. The second study included 11 patients and reported increased saliva production (1.74 vs 1.61 g/2 min baseline, p=0.029) after 24 weeks of therapy and decreased lymphocytic foci in total (but not lymphocytic foci per mm^2^); one patient developed lupus-like cutaneous lesions.^25^

#### Anakinra

A small RCT that randomised 26 patients (13 to anakinra and 13 to PLA) found no significant reduction in fatigue in the primary endpoint^14^ (comparison of fatigue scores at week 4, p=0.19); 2 patients experienced severe side effects (injection site reaction and gastroenteritis, respectively), 2 had a transient episode of neutropenia and 7 (54%) mild injection site reactions.

#### Baminercept

St Clair *et al*^18^ have recently reported on the clinical efficacy and safety of baminercept in 52 patients with primary-2002 randomised in a 2:1 ratio to receive subcutaneous injections of 100 mg of baminercept every week for 24 weeks or matching PLA. The primary end point was the change between screening and week 24 in the stimulated whole salivary flow (SWSF) rate. The change from baseline to week 24 in the SWSF rate did not differ significantly between the baminercept and the PLA groups (baseline-adjusted mean change −0.01 vs 0.07 mL/min; p=0.332). Baminercept was associated with a higher incidence of liver toxicity, including two serious adverse events.

#### Belimumab

The results reported by the Efficacy and Safety of Belimumab in Subjects with Primary Sjögren’s Syndrome (BELISS) open-label trial^57^ in 30 primary-2002 patients (all with systemic complications, early disease and/or abnormal biomarkers) showed that belimumab 10 mg/kg (weeks 0, 2 and 4, and then every 4 weeks until week 24) was associated with a higher rate of improvement in the composite outcome (improvement of at least two of the five following items: ≥30% reduction in VAS for dryness, fatigue, musculoskeletal pain and physician systemic activity, and ≥25% reduction in any of the B-cell activation biomarkers) in patients with early disease in comparison with those with systemic disease (73% vs 47%); the mean ESSDAI score decreased from 8.8 to 5.59 (p<0.0001) and the ESSPRI score from 6.44 to 5.56 (p=0.01). In the 19 patients who completed 1 year of treatment, a significant improvement in some ESSDAI involvements (glandular, lymphadenopathy and articular) was maintained.^22^ With respect to the safety profile, only one serious adverse event was reported (pneumococcal meningitis) after six drug infusions.

#### Epratuzumab

In 2006, a small prospective study including 15 patients with primary-2002 SjS^23^ reported a beneficial effect on VAS fatigue (<0.05), patient assessment (<0.05), physician assessment (<0.05) and tender joints (<0.05); five patients experienced severe adverse events (acute infusion reactions and infections).

#### Etanercept

Two studies (one RCT, one prospective) have been carried out in SjS patients; only the prospective study included primary-2002 patients and showed no significant improvements in the main sicca signs and symptoms.^29^

#### Infliximab

A prospective open-label study in 16 patients found significant improvements in subjective and objective measures after the administration of INF, although recently the authors have retracted the manuscript.^58^ In 2004, Mariette *et al*^12^ conducted an RCT including 103 patients and found no significant differences in the primary outcome, defined as improvement in at least 30% of the joint pain, fatigue and dryness VAS at 22 weeks (INF 20.4% vs PLA 16.7%, p=0.62) or in the majority of secondary outcomes (symptoms, salivary flow rates, ocular tests, QoL and salivary biopsy), with improvement only in fatigue and some analytical parameters in comparison with PLA.

#### Rituximab

Rituximab has been tested in three prospective cohort studies,^33–35^ one case-control study^36^ and four RCTs.^10 13 15 19^ A summary of the significant improvements reported with respect to baseline in the prospective cohort studies showed improvement in VAS for dryness,^34 36^ fatigue^34–36^ and pain/tender point count,^34^ while no significant improvements were reported for objective oral and ocular tests (except in the study by Pijpe *et al*^33^ in a subset of patients). In the case-control study, Carubbi *et al*^36^ compared the therapeutic effect of rituximab (n=22) and conventional immunosuppressive therapy (n=19) and found a significant improvement in patients treated with rituximab in the ESSDAI (<0.05), global VAS (<0.05), fatigue VAS (<0.01), dryness VAS (<0.01), physician VAS (<0.05), uSF (<0.01) and Schirmer test (<0.05) at the end of follow-up (120 weeks); the authors reported a complete lack of reported adverse events in either of the two arms in spite of the long-term nature of the study. With respect to the two small RCTs, Dass *et al*^13^ randomised 17 primary-2002 patients to receive rituximab (n=8) or PLA (n=9) and found no significant results in the primary outcome (improvement >20% VAS fatigue at 6 months, rituximab 87% vs PLA 56%, p=0.36), while Meijer *et al*^10^ randomised 30 primary-2002 patients to receive rituximab (n=20) or PLA (n=10) with no significant results for the primary outcome (improvement in SWSF rate at 48 weeks, p>0.05). Two large RCTs have recently been reported. In 2014, Devauchelle-Pensec *et al*^15^ randomised 122 primary-2002 patients to receive rituximab (n=63) or PLA (n=57) and found no significant results in the primary outcome (≥30 mm improvement at week 24 on at least 2 out of 4 VAS scores—dryness, fatigue, pain, global, 23% vs 22%, p=0.91), while Bowman *et al*^19^ randomised 133 primary-2002 patients to receive rituximab (n=67) or PLA (n=66) and found no significant results in the primary outcome (reduction ≥30% at week 48 in either fatigue or oral dryness VAS, rituximab 39.3% vs PLA 36.8%, p=0.76). Two recent meta-analyses including the four RCTs have confirmed the lack of significant between-group differences in mean improvements between baseline and week 24 values for fatigue VAS, oral dryness VAS, salivary flow rate and Schirmer test, and no significant difference between groups for the main adverse events.^59 60^

## Discussion

Current evidence on the therapeutic management of primary-2002 SjS patients is based on 9 RCTs (only four including ≥100 patients randomised to drug/PLA), 18 prospective cohort studies (all including ≤30 patients per study) and 5 case-control studies.

For oral topical therapies, available evidence is limited to five studies carried out in SjS patients (only one in primary-2002 patients) and one Cochrane SLR that assessed the management of dry mouth. Xerostomia is a subjective symptom with wide interindividual variation, and a satisfactory output of unstimulated whole saliva in one patient may lead another to complain of symptoms of dry mouth. All studies have shown significant within-group improvement in comparison with baseline subjective oral outcomes. Mechanical stimulation (chewing gum) was associated with increased saliva production in patients with residual capacity, but there is no evidence that gum may have more or less efficacy than saliva substitutes in reducing dry mouth symptoms. Due to the wide range of interventions, small trials (mean of 45 participants per trial), the RoB and the range of outcome measures for oral dryness, there is no strong evidence to support any specific intervention over another, and the conclusion is that, in the absence of an effective topical treatment, we recommend that the treatment of xerostomia should be directed towards improving patients’ complaints rather than increasing saliva production, including improvements in tooth health and the prevention of oral infections. The cost of long-term topical therapy is another important consideration, but there are no available studies on this issue. Other therapeutic interventions are under investigation. A recent double-blind, crossover-design study has evaluated the effects of intraoral electrostimulation,^61^ and further studies in primary-2002 patients are required to make a specific recommendation on its use.

For ocular topical therapies, among the 43 studies in SjS patients, only 6 (14%) were carried out in primary-2002 patients, while there are 4 SLRs on the management of dry eye using ATs, AS and plugs.^44 49 50 62^ The Cochrane SLR on AT showed that the AT formulations tested improved the signs and symptoms over the course of the studies included, although they found no consistent between-group differences when conducting head-to-head AT comparisons. The authors concluded that, given the large number of AT formulations compared and the wide variety of outcomes, it was difficult to propose that one over the counter AT formulation is superior to another for the treatment of dry eye syndrome. However, AT consistently improved ocular symptoms over the course of the trials included, based on within-group analyses, and three of four PLA-controlled trials consistently found that AT improved ocular symptoms compared with PLA (saline or vehicle), with a similar trend for many of the secondary outcomes. This review also found that the use of ATs is relatively safe, with the most common adverse events being blurred vision, ocular discomfort and foreign body sensation. It is important to consider that most outcomes were subjective measures of patient-reported outcomes rather than objective outcomes.^44^ With respect to non-steroidal anti-inflammatory drug (NSAID)/GC-based ocular tears, evidence not including primary-2002 SjS patients suggests careful use of tears containing NSAIDs or GCs due to the side effects associated with prolonged use.^63^ In these patients, ocular topical NSAIDs or corticosteroids may be a short-term therapeutic approach prescribed by ophthalmologists for the minimum time necessary (maximum 2–4 weeks).^63^

With respect to topical CyA, the pivotal study (a combination of two trials including 877 patients—270 fulfilled the 1993 SjS criteria, not detailed how many were primary or associated) evaluated 14 efficacy outcomes (4 objective, 10 subjective) with no definition of which were primary or secondary and found statistically significant differences between groups only in 4 (2 subjective and 2 objective). In spite of this, the FDA approved their use. In addition, a review of the results of all reported studies shows that most studies only demonstrated within-group improvements, and those that reported significant between-group differences (drug and vehicle) found improvement in only 20%–30% of the outcomes evaluated.^45^ In addition, a study extension trial found no additional improvement in subjective and objective measurements in patients treated for >6 months.^46^ Although SjS patients were included in variable proportions in the above-mentioned RCTs, there are no RCTs specifically in primary-2002 patients.^64^ Only one recent prospective study was made in primary-2002 patients, which found no significant differences between topical CyA and topical 0.1% FML.^40^ A recent small RCT using 0.03% tacrolimus tear drops found significant improvements in 24 primary-2002 patients^48^; the lack of definition of the population included (unclear whether they were primary or associated cases), the high rate of side effects and, especially, the limited number of cases does not support their widespread use in primary SjS patients, although they could be considered as a rescue therapy for CyA non-responders/intolerant patients.

With respect to AS and plug insertion, in addition to the very limited evidence available for primary-2002 patients, the two Cochrane SLRs found significant limitations in the quality of trials (incomplete descriptive statistics for reported treatment outcomes, sample sizes with too few participants, quasi-randomisation methods, complete masking may not have been feasible, short-term evaluation of efficacy, lack of details on adverse outcomes and tolerance). For AS, the Cochrane SLR concluded that current evidence suggests that 20% AS might provide some benefit in improving patient-reported symptoms over the short term (2 weeks), but longer periods of follow-up provide no evidence of improvement over longer periods, while no clear effects were found for objective clinical measures of the ocular surface. For punctal occlusion, the certainty of the evidence ranged from moderate to very low (frequently downgrading the level of evidence due to the high RoB or imprecision in effect estimates), and it was concluded that current evidence suggests that punctal plugs are a modestly effective means of treating dry eye. For all these reasons, the use of AS and plugs in primary SjS has been considered as a rescue therapeutic option, always under prescription by an ophthalmologist. A combination of AS and plugs has recently been evaluated^65^ in 28 SjS patients (primary and associated, criteria not detailed). Ophthalmologists play a key role in prescribing topical and systemic therapies for ocular dryness, always with a close multidisciplinary follow-up.

The two pivotal trials of pilocarpine included 629 patients with primary/associated SjS fulfilling the 1993 criteria and reported statistically significant differences between groups for the two primary VAS outcomes defined. With respect to cevimeline, the two pivotal trials included 272 patients with primary/associated SjS fulfilling the 1993 criteria and reported statistically significant differences between groups for the primary VAS outcomes. There is very limited evidence to support the use of these drugs in the treatment of oral dryness in primary-2002 patients (only one study using pilocarpine). It would seem appropriate to offer patients a trial of the drug, assuming there are no contraindications (ie, uncontrolled asthma, uncontrolled chronic obstructive pulmonary disease, uncontrolled cardiorenal syndrome, acute iritis, pregnancy, breast feeding) to the use of the drug. The trial should be prolonged, since the response can be delayed (up to 12 weeks), with a recommended dose of 5 mg three times a day to keep side effects to a minimum, since the adverse effects are dose dependent. Additional studies are required to clarify the role of muscarinic agonists in the treatment of xerostomia in primary SjS patients.

With respect to systemic synthetic immunosuppressive drugs, there are no new studies specifically assessing GCs in primary SjS in the last 20 years. For immunosuppressive agents, all reported studies (all including <20 patients) were principally centred on the efficacy in sicca features (with limited benefits) and laboratory parameters, with no specific analysis of the outcomes recommended in the guidelines (efficacy in systemic disease) and with an unacceptable rate of adverse events (41%–100%).^3^ In addition, there is a lack of head-to-head studies comparing the efficacy and safety profile of the different immunosuppressive agents.

The emergence of biological therapies has increased the therapeutic armamentarium available to treat SjS, but their use is limited by the lack of licensing. Available evidence is principally supported by the studies testing rituximab in primary SjS, making this drug the most frequently tested in these patients. There are six additional prospective cohort studies including ≤30 patients (one for etanercept, two—one extension—for belimumab, one for epratuzumab and two for abatacept), one small study (<30 patients) using anakinra, one medium-sized study (n=52) using baminercept and one large trial (>100 patients) using INF.

The results of trials on the efficacy of rituximab in the outcomes evaluated are summarised in the SLR published by Letaief *et al*.^59^ There were no statistically significant differences between groups for the primary outcomes based on subjective VAS dryness. The quality of evidence according to the Grades of Recommendation Assessment, Development and Evaluation (GRADE) approach was low for oral dryness, salivary flow rates, fatigue, mental health and ESSDAI meta-analysis and moderate for the Schirmer test meta-analysis. All studies included reported adverse effects, but with no significant differences between groups; the meta-analysis presented low-quality evidence according to the GRADE approach. The promising results obtained in small uncontrolled studies for the use of belimumab and abatacept should be confirmed in large RCTs before a solid therapeutic recommendation for use in primary SjS patients in standard clinical practice can be made. Limited data have been obtained from controlled trials to guide systemic treatment, although prospective studies using rituximab have suggested efficacy in systemic disease, especially in cryoglobulinemic vasculitis, as reported in unselected cryoglobulinemic patients.^66^

The current evidence supporting the efficacy and safety of the main topical therapeutic options for the treatment of sicca symptoms of primary SjS is solid but is extrapolated from the results of RCTs carried out in mixed populations of patients with dryness caused by SjS and other aetiologies. The overall low level of evidence from therapeutic studies specifically carried out in primary SjS patients fulfilling the current classification criteria underscores the need for considerably larger trials. In addition, there is no information on the differential efficacy and safety of the main systemic therapeutic options available and treatment-by-treatment choices will remain challenging in clinical practice.
